# The Buffering Effect of Mindfulness on Abusive Supervision and Creative Performance: A Social Cognitive Framework

**DOI:** 10.3389/fpsyg.2017.01588

**Published:** 2017-09-12

**Authors:** Xiaoming Zheng, Xin Liu

**Affiliations:** School of Economics and Management, Tsinghua University Beijing, China

**Keywords:** abusive supervision, creative performance, self-efficacy, mindfulness, social cognitive framework

## Abstract

Our research draws upon social cognitive theory and incorporates a regulatory approach to investigate *why* and *when* abusive supervision influences employee creative performance. The analyses of data from multiple time points and multiple sources reveal that abusive supervision hampers employee self-efficacy at work, which in turn impairs employee creative performance. Further, employee mindfulness buffers the negative effects of abusive supervision on employee self-efficacy at work as well as the indirect effects of abusive supervision on employee creative performance. Our findings have implications for both theory and practice. Limitations and directions for future research are also discussed.

## Introduction

Creative performance, which is conceptualized as the development of useful and novel ideas regarding procedures, products, or services (Amabile et al., 1996; Zhou and George, 2001; Zhou, 2003), plays a pivotal role in organizations. In today’s dynamic environments, organizations are heavily dependent on employee creative performance as a resource that enables them to respond to unforeseen challenges and to maintain competitive advantages (Tierney et al., 1999; Kijkuit and Van Den Ende, 2007; Shalley et al., 2009). Given the critical importance of creative performance, scholars have sought to explore its antecedents and found related leader behaviors, such as transformational leadership (Shin and Zhou, 2003) and empowering leadership (Zhang and Bartol, 2010), serve as primary drivers for employee creative performance (see Zhou and Shalley, 2003; Shalley and Gilson, 2004; Anderson et al., 2014; Zhou and Hoever, 2014, for reviews). In recent studies, researchers have shifted their attention from those positive leader behaviors to negative leader behaviors, examining the impact of abusive supervision—defined as leaders’ “sustained display of hostile, verbal and non-verbal behaviors, excluding physical contact” (Tepper, 2000, p. 178)—on creative performance (Liu et al., 2012; Zhang et al., 2014).

Nevertheless, what is less clear in both theory and practice is *why* and *when* abusive supervision influences employee creative performance. To date, only two studies have examined the relationship between abusive supervision and creative performance. Specifically, Liu et al. (2012) investigated the trickle-down effect of department leader abusive supervision on employee creativity by studying team leader abusive supervision as a mediator, and Zhang et al. (2014) unpacked the mediating role of intrinsic motivation and the moderating role of core self-evaluation to explain the relationship between abusive supervision and employee creativity. In view of the minimal extant research in this area, more studies are warranted that enrich the literature by developing an integrated theoretical model and exploring other mediating mechanisms as well as boundary conditions (Liu et al., 2012; Zhang et al., 2014). From a practical sense, creative performance is critical for organizations’ competitive advantage (Tierney et al., 1999; Kijkuit and Van Den Ende, 2007; Shalley et al., 2009), and abusive supervision is a very common phenomenon that has dysfunctional effects on organizations (for reviews, see Tepper, 2007; Martinko et al., 2013; Zhang and Liao, 2015; Mackey et al., 2017). In turn, managers are highly concerned about how to buffer the detrimental effects of abusive supervision on creative performance.

In our research, we use social cognitive theory (Bandura, 1986) as an overarching theoretical framework to advocate that self-efficacy at work, representing an individual’s belief in his or her capability to perform work activities with skill (Spreitzer, 1995; Bandura, 1997), is the underlying process explains why abusive supervision impairs employee creative performance. Social cognitive theory is one of the most influential theories in understanding individuals’ behaviors in certain social contexts (Bandura, 1997; Smith and Hitt, 2005). It argues that self-efficacy is changeable and malleable in various social contexts such as workplace, which in turn plays a key role in shaping individuals’ behaviors, especially when those individuals are performing tasks imbued with uncertainty (Bandura, 1997). Moreover, in the existing creative performance research, most studies have primarily adopted an intrinsic motivation approach but ignored other perspectives. To address this omission, scholars have called for attention to other theoretical frameworks as a means to extend the creative performance literature (Zhou and Shalley, 2008). Considering that the nature of creative performance relies on the individual’s confidence in his or her ability to confront various challenges (Shalley et al., 2004), it is reasonable to explore creative performance from a social cognitive perspective (Liao et al., 2010). Our research, therefore, builds on social cognitive theory to explicate *how* abusive supervision inhibits employee creative performance through self-efficacy at work.

Furthermore, integrating a regulatory approach, we propose that mindfulness is a moderator that buffers the detrimental effects of abusive supervision on employee creative performance as well as the underlying mechanism. Mindfulness is a psychological construct referring to “awareness and observation of the present moment without reactivity or judgment” (Glomb et al., 2011, p. 116). Emerging studies have suggested that the central function of mindfulness is to improve self-regulation over thoughts, emotions, and behaviors (Allen and Kiburz, 2012; Hülsheger et al., 2013, 2014, 2015; Leroy et al., 2013). According to Glomb et al. (2011), mindfulness encompasses two fundamental elements: (1) decoupling of the self from the experience and (2) decreased automaticity and reactivity. In essence, individuals with a high level of mindfulness can regulate their cognitions by detaching themselves from the experienced event and preventing themselves from having reactive responses to the event. When they encounter adverse events in the workplace, employees with a high level of mindfulness can separate the ego from those experiences and thereby avoid negative impacts both on their cognitions related to the self and on workplace behaviors (Long and Christian, 2015). Therefore, based on a regulatory approach, we argue that mindfulness can mitigate the detrimental impact of abusive supervision on self-efficacy at work and, in turn, creative performance.

**FIGURE 1:**
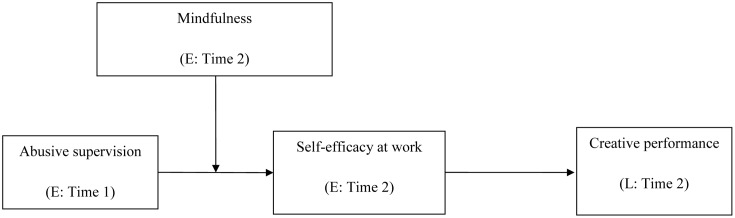
The theoretical model. *Note*: E = employee-rated variables, L = leader-rated variable.

Our research makes three primary theoretical contributions to the literature. First, by adopting a perspective based on social cognitive theory, we advance the research by identifying a new underlying mechanism that explains the relationship between abusive supervision and creative performance. In the extant literature, little effort has been exerted to link negative leader behaviors to creative performance. Using social cognitive theory as the overarching theoretical framework, our research posits that employee self-efficacy at work mediates the relationship between abusive supervision and employee creative performance. This understanding adds to our knowledge of *why* abusive supervision impacts employee creative performance, and consequently enriches the literature of abusive supervision and creative performance. Second, drawing on a regulatory approach, we contribute to this area of research by documenting a new boundary condition that mitigates the detrimental impacts of adverse events on employees’ cognitions and behaviors. This work integrates the regulatory approach with social cognitive theory, and also enlarges the scope of social cognitive theory as well as the abusive supervision literature. Third, our research expands the mindfulness literature by unveiling its critical role in buffering the negative consequences of abusive supervision. Indeed, despite emerging studies that address the regulation function of mindfulness at work (e.g., Hülsheger et al., 2013, 2014; Michel et al., 2014), Glomb et al. (2011) have called for further examination of the role of mindfulness in the workplace. Our research responds to this call and, in so doing, extends the literature. **Figure 1** depicts our theoretical model.

## Theoretical Groundings and Hypotheses Development

In extant research, leader behaviors have been recognized as a critical contextual factor that impacts employee creative performance (Zhou and Shalley, 2003; Anderson et al., 2014; Zhou and Hoever, 2014). For example, transformational leadership (Shin and Zhou, 2003; Gong et al., 2009), benevolent leadership (Wang and Cheng, 2010), and empowering leadership (Zhang and Bartol, 2010) have been identified as means to foster creative performance in the workplace. Most of this research has focused on positive leader behaviors, but some recent studies have explored the impacts of negative leader behaviors on creative performance. A salient example is the focus on abusive supervision (e.g., Liu et al., 2012; Zhang et al., 2014). In addition, a growing body of research has found that exposure to abusive supervision can lead to dysfunctional outcomes such as reductions in job satisfaction, organizational commitment, psychological well-being, job performance, and organizational citizenship behaviors (Tepper, 2007; Liu et al., 2010; Martinko et al., 2013; Zhang and Liao, 2015; Mackey et al., 2017). In total, the evidence supports the existence of a negative relationship between abusive supervision and creative performance.

### The Mediating Role of Self-efficacy at Work

Drawing from social cognitive theory (Bandura, 1986), our research proposes that abusive supervision undermines employee creative performance by decreasing employee self-efficacy at work. Bandura (1997) posits that self-efficacy is a major cognitive mechanism that integrates information to instruct individual behaviors. It is constructed from four sources of information: enactive mastery experience, vicarious experience, social persuasion, and physiological state. Enactive mastery experience refers to an individual’s past performance, which serves as an indicator of capability; vicarious experience represents the phenomenon in which individuals change their efficacy beliefs by observing and learning from role models; social persuasion refers to others’ verbal persuasion that confirms the individual’s competencies; and physiological state comprises the individual’s affective states related to performing tasks. These four sources of information have been well documented in fields such as athletic attainment, clinical dysfunction, education, and health promotion (Bandura, 1997, 2001). Extending the theory to the organizational context, we argue that abusive supervision, as a commonly encountered phenomenon in the workplace, will diminish the effectiveness of these information sources and consequently inhibit employee self-efficacy at work.

Abusive supervision refers to subordinates’ perceptions regarding leaders’ engagement in sustained hostile, verbal and non-verbal behaviors, excluding physical abuse (Tepper, 2000). Abusive supervisors often ridicule, yell at, and intimidate employees; they do not acknowledge employees’ achievements or may even take credit for them; and they attribute unfavorable outcomes to employees (Tepper et al., 2009). We posit that abusive supervision has detrimental effects on all four sources of employee self-efficacy at work. Specifically, abusive supervisors are very likely to laugh at employees or undervalue employees’ contributions, which may act as a source of negative social persuasion and impair employee self-efficacy at work. Abusive supervisors also tend to ignore employees’ past accomplishments or make negative remarks to employees. These behaviors may frustrate employees, destroy their mastery experiences, and wreck beliefs in their own competencies, especially when they previously felt entitled to those beliefs (Lian et al., 2012; Harvey et al., 2014). In addition, abusive supervision may lead to negative affective states in employees such as anxiety, depression, and hostility (Tepper, 2000; Lian et al., 2014; Mackey et al., 2017); these negative physiological states may subsequently destroy the development of self-efficacy at work (Bandura, 1997). Finally, employees usually observe and learn from social models as a means to improve their abilities and skills for managing workload and ultimately developing personal efficacy. In the workplace, leaders are often regarded as role models for employees (Salancik and Pfeffer, 1978; Mayer et al., 2009; Liu et al., 2012). However, subordinates who operate in an environment characterized by abusive supervision cannot learn from their leaders in a positive way. Because abusive supervision may likewise undermine coworkers’ performance (Peng et al., 2014; Mitchell et al., 2015), employees may not be able to learn positive lessons from their coworkers, either; in a more positive environment, they might be able to gain effective abilities and develop a sense of self-efficacy at work through their coworkers’ influence.

Thus, abusive supervision damages all four sources of information of self-efficacy at work—that is, enactive mastery experience, vicarious experience, social persuasion, and physiological state—and harms employees’ belief in their own capabilities. Therefore, we hypothesize that:

Hypothesis 1: Abusive supervision is negatively related to employee self-efficacy at work.

Social cognitive theory (Bandura, 1997) also emphasizes the central and pervasive role of self-efficacy in shaping agentic behaviors. Specifically, Bandura (2001) argued that the main features of personal agency include intentionality, forethought, self-reactiveness, and self-reflectiveness. Intentionality means that individuals intentionally set goals and make plans; forethought suggests that individuals anticipate the outcomes of prospective actions and take actions to generate favorable consequences; self-reactiveness indicates that individuals regulate the execution process and use it to shape appropriate actions; and self-reflectiveness argues that individuals will reflect on the self and examine their own functioning. Underlying these core features of personal agency, individuals’ beliefs in their capabilities are the key to their perception of control over their own actions and external environments (Bandura, 2001): “Unless people believe they can produce desired results and forestall detrimental ones by their actions, they have little incentive to act or to persevere in the face of difficulties. Whatever other factors may operate as guides and motivators, they are rooted in the core belief that one has the power to produce effects by one’s actions” (p. 10).

Considering that creative performance is usually accompanied by obstacles and uncertainty, and requires persistent devotion of great effort to achieve the desired performance (Amabile et al., 1996; Oldham and Cummings, 1996), we argue that self-efficacy occupies a key role in the realization of creative performance. Individuals with higher self-efficacy at work are more likely to generate novel and useful ideas that achieve the established goals and to stick to those goals even in the face of difficulties (Bandura, 1986). This argument has received support from a number of previous studies. For instance, Barron and Harrington (1981) argued that self-confidence is a crucial characteristic that is linked to employee creativity. Tierney and Farmer (2004) showed that employees with high self-efficacy will proactively propose creative solutions in their work. Recently, Liao et al. (2010) also found that self-efficacy is positively related to employee creative performance.

Based on this findings, and using social cognitive theory as an overarching theoretical framework to integrate the abusive supervision and creative performance literatures, we posit that abusive supervision impairs employee self-efficacy at work, which in turn harms employee creative performance. Therefore, we propose that:

Hypothesis 2: Employee self-efficacy at work mediates the relationship between abusive supervision and creative performance.

### The Moderating Role of Mindfulness

Mindfulness is a psychological construct related to attention and awareness of the present moment without judgment or reactivity (Brown and Ryan, 2003; Baer et al., 2006; Glomb et al., 2011). It is rooted in Eastern spirituality, especially Buddhism (Marques, 2011; Purser and Milillo, 2014), and has garnered increasing attention in academic research (Allen and Kiburz, 2012; Leroy et al., 2013; Sutcliffe et al., 2016). In organizational studies, emerging studies have shed light on the role of mindfulness in the workplace (e.g., Fiol and O’Connor, 2003; Levinthal and Rerup, 2006; Weick and Putnam, 2006; Weick and Sutcliffe, 2006; Dane, 2011; Ray et al., 2011).

Mindfulness can vary from person to person (Brown and Ryan, 2003). Its most readily evident feature is the self-regulation function, through which “mindfulness and mindfulness-based practices lead to improved self-regulation and, ultimately, higher functioning” (Glomb et al., 2011, p. 124). More specifically, Glomb et al. (2011) developed a theoretical model that identified two core mental processes underlying mindfulness. On the one hand, individuals with a high level of mindfulness can induce “a decoupling of the self (i.e., ego) from events, experiences, thoughts, and emotions” (p. 124), which makes them detached and reduces self-relevance inference tendencies. On the other hand, individuals with a high level of mindfulness will experience “a decrease in automaticity of mental processes in which past experiences, schemas, and cognitive habits constrain thinking” (p. 124), which reduces narrow thought (Hafenbrack et al., 2014) and defuses reactive responses to adverse events. Through these dual processes, mindfulness can enable individuals to “stay in the moment” and to not evaluate or react to the events or experiences occurring at that time, which will enhance their self-regulatory functions and defuse the dysfunctional impacts of adverse events.

Accordingly, based on a regulatory approach, we propose that mindfulness can buffer the detrimental impacts of abusive supervision on employee self-efficacy at work. As mentioned earlier, abusive supervisors who routinely criticize or ridicule employees will impair employee self-efficacy in a self-oriented and automatic manner. When employees have a high level of mindfulness, however, they can decouple themselves from those adverse experiences. Rather than perceiving those experiences in relation to the self, mindful employees can take an objective view toward the experiences. Moreover, employees with a high level of mindfulness can reduce the automaticity of self-doubt and regulate their cognitive responses to abusive supervision. By comparison, employees with a low level of mindfulness are more likely to experience decreased self-efficacy at work in the wake of abusive supervision, as they are more likely to take the adverse experiences personally and, in turn, to doubt their own capabilities in accomplishing job tasks. A recent study from Long and Christian (2015) also noted that mindfulness mitigates the effect of an event involving injustice on rumination—evidence that supports our argument. In keeping with this line of reasoning, we predict that:

Hypothesis 3: Mindfulness moderates the relationship between abusive supervision and employee self-efficacy at work, such that the relationship is weaker for those high rather than low in mindfulness.

### The Moderated Mediation Model

Incorporating a regulatory approach into the social cognitive perspective, our research proposes an integrated model in which mindfulness moderates the mediating mechanism for self-efficacy at work in the relationship between abusive supervision and creative performance. Employees with high levels of mindfulness can detach themselves from and reduce their automatic reactions to adverse stimuli—in this case, abusive supervision—in the workplace. When they possess such a self-regulation function, mindful employees can maintain their self-efficacy at work in the face of abusive supervision, thereby remaining creative in the workplace. In contrast, employees with low levels of mindfulness are more likely to be hurt by abusive supervision, lose faith in their own capabilities to finish job tasks, and therefore be less creative in the workplace. Summarizing these relationships, we posit that:

Hypothesis 4: Mindfulness moderates the mediating effect of employee self-efficacy at work on the relationship between abusive supervision and creative performance, such that the mediating effect is weaker for those high rather than low in mindfulness.

## Materials and Methods

### Sample and Procedures

We collected data from a company located in northern China. The participants were frontline technicians who worked in teams to manufacture various components of electrical equipment. In the company, employees were encouraged to be creative so as to solve work-related issues with novel and useful ideas. Our research plan had the full support of the company’s top management team and also received assistance from the human resources department. To counter the potential threat of common method bias (Podsakoff et al., 2003, 2012), we collected data from two different sources (i.e., leaders and employees) across two time points (i.e., time 1 and time 2). To enhance data quality, our research team prepared the paper-based questionnaires in advance, brought them to the conference room in the company, and collected the completed questionnaires on site. In addition, we explained our research purposes to the participants and assured them of the confidentiality of their responses.

A total of 98 direct leaders and 600 frontline employees were invited to participate in our research. At time 1, employees were asked to rate the level of abusive supervision they experienced and their own intrinsic motivation (as a controlled mediator). In total, we received 578 responses, representing a response rate of 96.33%. At time 2, 6 months later, employees who had completed the time 1 survey were invited to assess their own mindfulness and self-efficacy at work, while direct leaders were asked to evaluate their subordinates’ creative performance. In this wave, 355 employees (response rate = 61.42%) and 82 leaders (response rate = 83.67%) rating 331 employees returned the completed surveys. In addition, demographic information, including gender, age, education, and tenure with leader, was provided by the human resources department with the participants’ permission.

After matching responses from employees and leaders based on identification codes assigned to them, the final sample consisted of 287 employees and 79 leaders. Among all employees, the average age was 25.44 years old (*SD* = 5.19) and the average tenure with leader was 1.27 years (*SD* = 1.06). Males accounted for 66% of the participants, and 48.43% of the employees had a junior college or higher education degree.

### Measures

Given that all measures were original in English, standard translation and back-translation procedures were followed (Brislin, 1986).

#### Abusive Supervision

It was measured with Tepper’s (2000) 15-item scale. Employees were asked to indicate how often their direct leader engaged in behaviors such as “He/she tells me my thoughts or feelings are stupid” and “He/she makes negative comments about me to others.” A five-point Likert-type scale ranging from 1 (*Never*) to 5 (*Very often*) was used (Cronbach’s alpha [α] = 0.97).

#### Self-efficacy at Work

It was assessed with a three-item scale developed by Spreitzer (1995) who used the “competence” subdimension to represent self-efficacy specific to work. Sample items included “I am confident about my ability to do my job” and “I am self-assured about my capabilities to perform my work activities.” A seven-point Likert scale, ranging from 1 (*strongly disagree*) to 7 (*strongly agree*), was used (α = 0.93).

#### Mindfulness

It was evaluated with the 15-item Mindful Attention and Awareness Scale developed by Brown and Ryan (2003), which has been widely used to measure mindfulness traits in the general population (Hülsheger et al., 2013, 2014). Sample items included “I tend to walk quickly to get where I am going without paying attention to what I experience along the way” (reverse scored) and “I rush through activities without being really attentive to them” (reverse scored). A six-point Likert-type scale was adopted for this scale, ranging from 1 (*almost never disagree*) to 6 (*almost always*) (α = 0.89).

#### Creative Performance

It was rated with a three-item scale developed by Oldham and Cummings (1996). This scale asked the leader to evaluate his or her agreement on the following descriptions for the specific employee: “The work he/she produces is original and practical (original and practical work refers to developing ideas, methods, or products that are both totally unique and especially useful to the organization),” “The work he/she produces is adaptive and practical (adaptive and practical work refers to using existing information or materials to develop ideas, methods, or products that are useful to the organization),” and “The work he/she produces is creative (creativity refers to the extent to which the employee develops ideas, methods, or products that are both original and useful to the organization).” A seven-point Likert-type scale was used, ranging from 1 (*strongly disagree*) to 7 (*strongly agree*) (α = 0.78).

#### Control Variables

We controlled for employees’ gender, age, education, and tenure with the leader because previous studies have shown that these demographic variables are related to abusive supervision and employee creative performance (Zhang and Bartol, 2010). More importantly, given that intrinsic motivation has been identified as a mechanism that transmits the influence of abusive supervision on employee creative performance (Zhang et al., 2014), we regarded it as a controlled mediator and empirically examined whether the social cognitive framework (i.e., self-efficacy) exerted an incremental effect beyond the motivational perspective (i.e., intrinsic motivation). Intrinsic motivation was rated with three items used by Zhang and Bartol (2010), such as “I enjoy finding solutions to complex problems.” A five-point Likert-type scale was adopted, ranging from 1 (*strongly disagree*) to 5 (*strongly agree*) (α = 0.78).

### Analytical Strategy

First, we performed confirmatory factor analyses (CFAs) to examine the discriminant validity of our key variables, including abusive supervision, self-efficacy, mindfulness, and creative performance. Second, considering the nested nature of our data, we used multilevel structural equation modeling (MSEM) method with Mplus to analyze our data (Preacher et al., 2010). Third, we adopted the method suggested by Preacher and Selig (2010) to test the mediation hypothesis (i.e., Hypothesis 2). Finally, we used Monte Carlo simulation with 20,000 replications to estimate the 95% confidence intervals (CIs) of the conditional indirect effect (Bauer et al., 2006), thus testing the moderated mediation hypothesis (i.e., Hypothesis 4).

## Results

**Table 1** presents the means, standard deviations, correlations, reliabilities, data sources, and collection time for all of the variables. As expected, abusive supervision was negatively correlated with self-efficacy (*r* = -0.17, *p* < 0.01) and creative performance (*r* = -0.14, *p* < 0.05). Meanwhile, self-efficacy was positively correlated with creative performance (*r* = 0.17, *p* < 0.01).

**Table 1:** Variable means, standard deviations, correlations, reliabilities, data sources, and collection schedule^a^.

Variables	Mean	*SD*	1	2	3	4	5	6	7	8	9
1. Gender	0.66	0.48									
2. Age	25.44	5.19	-0.10								
3. Education	3.63	0.86	-0.14*	0.13*							
4. Tenure with leader	1.27	1.06	-0.04	0.18**	0.01						
5. Intrinsic motivation (T1)	3.78	0.60	-0.06	0.12*	0.24***	0.09	***(0.78)***				
6. Abusive supervision (T1)	1.49	0.79	0.09	-0.11	-0.16**	-0.04	-0.03	***(0.97)***			
7. Mindfulness (T2)	4.80	0.62	-0.08	0.24***	0.11	0.02	0.07	-0.14*	***(0.89)***		
8. Self-efficacy at work (T2)	5.55	0.92	-0.04	0.18**	0.24***	0.07	0.24***	-0.17**	0.35***	***(0.93)***	
9. Creative performance (T2)	5.18	0.94	0.13*	-0.04	0.18**	-0.01	0.01	-0.14*	0.09	0.17**	***(0.78)***


**^a^n* = 287 at the individual level, *n* = 79 at the team level. Data for variables 1–4 were obtained from the human resources department; data for variables 5 and 6 were reported by employees at time 1; data for variables 7 and 8 were reported by employees at time 2; data for variable 9 were rated by the leader at time 2. For gender, female = 0, male = 1. For education, primary school degree = 1, secondary school degree = 2, vocational school degree = 3, junior college degree = 4, undergraduate degree = 5, graduate degree = 6. Cronbach’s alpha values for the variables are shown in italics along the diagonal in the brackets. ^∗^*p* < 0.05, ^∗∗^*p* < 0.01, ^∗∗∗^*p* < 0.001.*

### Preliminary Analyses

We performed confirmatory factor analyses (CFA) to ensure the discriminant validity of the measures. Considering that the ratio of sample size and total item numbers may potentially impair fit indexes, we created three-item parcels for the mindfulness variable following the item-to-construct-balance method (Williams et al., 2009). The CFA results were summarized in **Table 2**. They indicated that, compared with the alternative models, the hypothesized four-factor model best fitted the data [χ^2^(246) = 601.71, *p* < 0.001; SRMR = 0.03, RMSEA = 0.07, TLI = 0.94, CFI = 0.95]. Therefore, measures of the studied variables in our research had good discriminant validity.

**Table 2:** Results of confirmatory factor analysis^a^.

Models	χ^2^	*df*	Δχ^2^	*Δdf*	SRMR	TLI	CFI	RMSEA
**Four-factor model:**								
The hypothesized model	601.71	246			0.03	0.94	0.95	0.07
**Three-factor model:**								
Combine self-efficacy and mindfulness	1086.29	249	484.58^∗∗∗^	3	0.07	0.85	0.87	0.11
Combine self-efficacy and creative performance	853.28	249	251.57^∗∗∗^	3	0.07	0.89	0.90	0.09
Combine creative performance and mindfulness	864.98	249	263.27^∗∗∗^	3	0.07	0.89	0.90	0.09
**Two-factor model:**								
Combine self-efficacy, mindfulness, and creative performance	1496.40	251	894.69^∗∗∗^	5	0.10	0.78	0.80	0.13
**One-factor model:**								
Combine all	2130.50	252	1528.79^∗∗∗^	6	0.14	0.67	0.70	0.16


*^a^Both Δ*χ*^2^ and Δ*df* were compared with the hypothesized four-factor model. ^∗∗∗^*p* < 0.001.*

### Hypotheses Testing

#### Test of Main Effect

Hypothesis 1 predicts that abusive supervision is negatively related to employee self-efficacy at work. As shown in **Table 3**, abusive supervision was negatively associated with employee self-efficacy at work in our study (*B* = -0.24, *SE* = 0.09, *p* < 0.05; Model 1). Therefore, Hypothesis 1 was supported.

**Table 3:** Results for employee self-efficacy at work and employee creative performance^a^.

Variables	Self-efficacy at work				Creative performance			
		
	M1		M2		M3		M4	
				
	*B*	*SE*	*B*	*SE*	*B*	*SE*	*B*	*SE*
*Intercept*	4.54***	0.38	4.47***	0.33	4.62***	0.42	4.61***	0.40
**Controls**								
Gender	0.03	0.12	0.07	0.10	0.20	0.11	0.21	0.11
Age	0.02*	0.01	0.01	0.01	-0.01	0.01	-0.01	0.01
Education	0.19**	0.06	0.19***	0.05	0.12	0.08	0.12	0.08
Tenure with leader	0.03	0.05	0.05	0.05	-0.05	0.04	-0.04	0.04
Intrinsic motivation					-0.02	0.09	-0.01	0.09
**Predictors**								
Abusive supervision	-0.24*	0.09	-0.15*	0.07	-0.04	0.06	-0.04	0.06
Mindfulness			0.41***	0.10			0.12	0.08
Abusive supervision × Mindfulness			0.48***	0.11			0.05	0.10
Self-efficacy at work					0.18*	0.07	0.14*	0.07


**^a^n* = 287 at the individual level, *n* = 79 at the team level. Unstandardized coefficients were presented. ^∗^*p* < 0.05, ^∗∗^*p* < 0.01, ^∗∗∗^*p* < 0.001.*

#### Test of Mediation Effect

Hypothesis 2 proposes that employee self-efficacy at work mediates the relationship between abusive supervision and employee creative performance. The results presented in **Table 3** showed that employee self-efficacy at work was positively associated with creative performance (*B* = 0.18, *SE* = 0.07, *p* < 0.05; Model 3) after controlling for the effect of abusive supervision. Further, we used Preacher and Selig’s (2010) method to estimate confidence intervals (*CI*) of this mediation effect. The results showed that the indirect effect of abusive supervision on employee creative performance via employee self-efficacy at work was significant (estimate = -0.04, 95% *CI* = [-0.08, -0.01]). Therefore, Hypothesis 2 was supported.

#### Test of Moderation Effect

**FIGURE 2:**
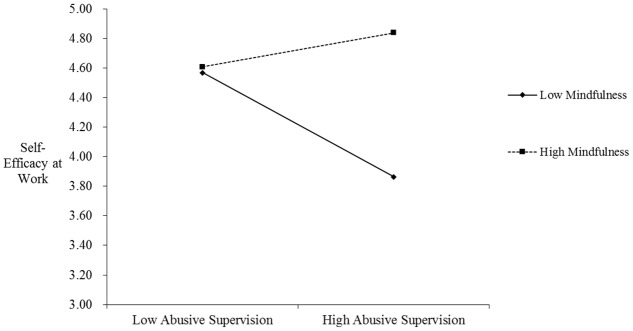
The moderating effect of mindfulness on the relationship between abusive supervision and self-efficacy at work.

Hypothesis 3 posits that mindfulness moderates the relationship between abusive supervision and employee self-efficacy at work such that the relationship is weaker for those high—but not low—in mindfulness. As shown in **Table 3**, the interaction term of abusive supervision and mindfulness was significantly related to employee self-efficacy at work (*B* = 0.48, *SE* = 0.11, *p* < 0.001; Model 2). The interaction plot (see **Figure 2**) and simple slope tests showed that when the level of mindfulness was low (1 *SD* below the mean), the relationship between abusive supervision and employee self-efficacy at work was significantly negative (simple slope *B* = -0.48, *p* < 0.001); in contrast, when the level of mindfulness was high (1 *SD* above the mean), the relationship between abusive supervision and employee self-efficacy at work became non-significant (simple slope *B* = 0.15, *p* = 0.16, *n.s.*). These results revealed that mindfulness buffered the impact of abusive supervision on employee self-efficacy at work, so Hypothesis 3 was supported.

#### Test of Moderated Mediation Model

Hypothesis 4 advances a moderated mediation model, arguing that mindfulness moderates the mediating effect of employee self-efficacy at work on the relationship between abusive supervision and creative performance, such that the mediating effect is weaker for those high—but not low—in mindfulness. The results of Monte Carlo simulation with 20,000 replications in **Table 4** showed that the indirect effect of abusive supervision on creative performance via self-efficacy at work was significant (*B* = -0.06, 95% *CI* = [-0.13, -0.003]) for low level of mindfulness (1 *SD* below the mean), but was non-significant (*B* = 0.02, 95% *CI* = [-0.002, 0.05]) for high level of mindfulness (1 *SD* above the mean). Moreover, the difference between low and high levels of mindfulness for the indirect effect was significant (*B* = 0.08, 95% *CI* = [0.003, 0.16]). Therefore, Hypothesis 4 was supported.

**Table 4:** Results of the conditional indirect effects^a^.

Conditions	Abusive supervision (X) → Self-efficacy at work (M) → Creative performance (Y)	
	
	Indirect Effect	[95% Confidence Interval]	
Low employee mindfulness (-1 *SD*)	-0.06	-0.13	-0.003
High employee mindfulness (+1 *SD*)	0.02	-0.002	0.05
Differences between low and high conditions	0.08	0.003	0.16


**^a^n* = 287 at the individual level, *n* = 79 at the team level.*

### Supplementary Analyses

To rule out the possibility that abusive supervisors might rate employees as being lower performing in any area, we ran supplementary analyses using job performance as our dependent variable. We invited the supervisor to evaluate employees’ job performance using the five-item scale from Podsakoff and MacKenzie (1989, Unpublished). A sample item is “This employee always completes the duties specified in his/her job description” (α = 0.73; scale ranging from 1 = strongly disagree to 7 = strongly agree). We followed the same procedures to test the mediation effect of self-efficacy at work in linking abusive supervision and employee job performance. The results showed that self-efficacy at work was not significantly related to employee job performance (*B* = 0.04, *SE* = 0.06, *p* = 0.51, *n.s.*) while controlling for the effect of abusive supervision. Therefore, the obtained effects seem to be unique to creative performance ratings.

## Discussion

In this study, we explored explanations of *why* and *when* abusive supervision impairs employee creative performance. Adopting social cognitive theory as an overarching theoretical lens, we argued that employee self-efficacy at work is the mediating mechanism underlying the relationship between abusive supervision and employee creative performance. Furthermore, integrating social cognitive theory with a regulatory approach, we predicted that mindfulness is the boundary condition for the indirect effect of abusive supervision on employee creative performance via employee self-efficacy at work. Analyses of data collected at multiple time points and from multiple sources indicated that abusive supervision was negatively related to employee self-efficacy at work; self-efficacy at work mediated the relationship between abusive supervision and employee creative performance; employee mindfulness buffered the negative impact of abusive supervision on employee self-efficacy at work; and employee mindfulness further moderated the mediating mechanism of self-efficacy at work on the relation between abusive supervision and employee creative performance. Informed by these findings, our research has implications for theory and practice, and suggest avenues for future research.

### Theoretical Implications

Our research has several implications for theory. First, we contribute to the literatures on abusive supervision and creative performance by identifying a social cognitive path (i.e., self-efficacy at work) as an underlying mechanism that explains the relationship between abusive supervision and creative performance. In the past, relatively little effort has been made to uncover why abusive supervision impairs creative performance. Until now, only a motivational path (Zhang et al., 2014) has been identified in the literature as the explanatory mechanism, while other possible paths were left unexplored. As a consequence, adequate understanding of *why* abusive supervision impairs creative performance has been lacking (Liu et al., 2012). Drawing upon social cognitive theory, our research documents that abusive supervision harms employee self-efficacy at work, which in turn hurts employee creative performance. In addition, our identification of the negative impact of abusive supervision on self-efficacy at work broadens the outcome set of abusive supervision research (for reviews, see Tepper, 2007; Martinko et al., 2013; Zhang and Liao, 2015; Mackey et al., 2017).

Second, we advance the literatures on abusive supervision and creative performance by incorporating a regulatory approach and revealing mindfulness as a boundary condition that buffers the dysfunctional effect of abusive supervision on cognitions and behaviors. According to social cognitive theory, an individual’s personal belief in his or her capabilities is influenced by information gleaned from enactive mastery experiences, vicarious experiences, social persuasion, and physiological state. However, if their information sources are harmed by external factors (e.g., abusive supervision), individuals are more likely to lose faith in their own capabilities to finish job tasks, which consequently has negative impacts on their behaviors at work. Incorporating a regulatory approach into our research, we theorized that mindfulness can be an effective moderator that buffers such detrimental effects. In particular, our results indicated that abusive supervision may impair employee self-efficacy at work and subsequent creative performance only when mindfulness is low, rather than high. This finding not only identifies the boundary condition of abusive supervision effects (Liu et al., 2012; Zhang et al., 2014), but also extends the contingency of social cognitive theory (Bandura, 2001).

Third and relatedly, we enrich the mindfulness literature by examining mindfulness’s regulatory function in the face of negative leader behavior. In extant research, mindfulness is an emerging focus, but most of the studies to date have centered on its beneficial impacts on stress and well-being (see Chiesa and Serretti, 2010, for a review). Our research moves beyond the current view to examine the regulatory function of mindfulness at work (Hülsheger et al., 2013, 2014). By integrating a regulatory approach with social cognitive theory, we found that mindfulness buffers the detrimental effects of negative leader behavior (i.e., abusive supervision) on a cognitive factor (i.e., self-efficacy), and further reduces the indirect effects of abusive supervision on creative performance. These findings have broadened the nomological network of the mindfulness literature (Chiesa and Serretti, 2010; Glomb et al., 2011).

### Practical Implications

Our study also has implications for practice. First, our results reveal that abusive supervision impairs employee self-efficacy at work and subsequently decreases creative performance in the workplace. This outcome serves as a warning that abusive supervision is, indeed, detrimental in organizations (Liu et al., 2012). To avoid its negative effects, organizations should take actions and coach leaders to manage their abusive behaviors.

Second, our results show that employee self-efficacy at work acts as a mediating mechanism to transmit the detrimental consequences of abusive supervision. This finding sends a valuable message: Organizations should pay more attention to the development of employee self-efficacy at work. For example, they might regularly provide training programs to develop employee self-efficacy at work, which might help mitigate the detrimental effects of abusive supervision on employee creative performance.

Third, our research indicates that mindfulness plays an important role in buffering the negative impacts of abusive supervision on self-efficacy and creative performance. Considering that it is extremely difficult to change leader behaviors (Salancik and Pfeffer, 1978) and that abusive supervision is prominent in organizations (Tepper, 2000), we suggest organizations should promote mindfulness as a useful tool to counter negative leader behaviors such as abusive supervision. Previous studies have shown that mindfulness can be cultivated and enhanced by training techniques such as mindfulness meditation (Brown and Ryan, 2003). In this sense, organizations can consider to launch mindfulness training programs in the workplace to enhance both leaders’ and employees’ self-regulatory abilities (Glomb et al., 2011). At the same time, we encourage employees to exert efforts to improve their own level of mindfulness. By doing so, employees can shield themselves from the detrimental impacts of negative encounters such as abusive supervision while remaining creative—a key capability that may enable them to achieve career success in the workplace (Dries et al., 2008; Chen et al., 2015).

### Limitations and Future Research

Our research also has several limitations that provide avenues for future research. First, as with any field survey study, we cannot make causal inferences based on the results of our research. For example, the literature of victim precipitation (Olweus, 1978; Elias, 1986) suggests that low self-efficacy might potentially result in more supervisory abuse (Tepper et al., 2011). Our research attempted to address this issue by collecting data from multiple time points and multiple sources (Podsakoff et al., 2012). However, given the feasibility of our data collection process, we were unable to measure all variables at all time points. Thus, we suggest future studies adopt a longitudinal or laboratory experiment design to further test the causal relationships among the variables studied in our research (Cole and Maxwell, 2003; Little, 2013).

Second, creative self-efficacy—rather than general self-efficacy—may be a more appropriate mediator. According to social cognitive theory, self-efficacy is the core cognitive mechanism that drives behaviors in the workplace (Bandura, 1997, 2001), and self-efficacy at work fits well with the examination of how abusive supervision harms information sources of developing self-efficacy at work and consequently hurts creative performance. Nevertheless, creative self-efficacy has been found to be significantly related to creative performance (Tierney and Farmer, 2002). Therefore, we advise future research to examine the mediating role of creative self-efficacy, and to test whether it is a more critical mediator than self-efficacy at work. Abusive supervision may also influence creative performance via other processes such as emotions (Mawritz et al., 2014) and resources (Whitman et al., 2014). To test these possibilities, future research should explore other potential mediating mechanisms.

Furthermore, we suggest that scholars should pay more attention to the role of mindfulness in the workplace. In the extant research, studies are emerging that discuss the effects of mindfulness. For instance, Sutcliffe et al. (2016) presented a cross-level review of mindfulness in organizations, and Hyland et al. (2015) provided an overview of the definition and application of mindfulness in work settings. However, only a few studies have empirically examined the role of mindfulness at work (e.g., Hülsheger et al., 2013, 2014; Long and Christian, 2015). Given that our research found mindfulness was, indeed, useful to cope with negative experiences such as abusive supervision, we call for more studies to empirically test the effects of mindfulness in the workplace, thereby enriching our understanding of this relationship. Moreover, in addition to serving as a buffer against adverse events, mindfulness may have beneficial effects in the face of positive treatment. For example, mindful individuals may be more aware of their own strengths and weaknesses, and hence more likely to remain humble and objective when accorded external praise. Future studies might test this prediction and further explore other functions of mindfulness in the workplace.

Finally, the interaction plot in **Figure 2** indicates a possible performance-enhancing pathway, that is, high mindfulness employees may gain high self-efficacy at work and consequently high creative performance in face of adverse events such as abusive supervision (Tepper et al., 2017). Perhaps, when employees have a high level of mindfulness, they can reduce their automaticity of self-doubt and regulate their attention and focus to their job tasks. In this way, when encountering abusive supervision, they will not take the abuse personally; on the contrary, they may even try to prove their supervisor wrong by having high confidence about their ability to do jobs and by demonstrating better performance (i.e., creative performance). Future research could examine this possibility in greater depth.

## Conclusion

Drawing upon social cognitive theory and integrating a regulatory approach, our research uncovered why and when abusive supervision impairs employee creative performance. Our results indicate that employee self-efficacy at work is the mediating path underlying the relationship between abusive supervision and employee creative performance. Moreover, mindfulness buffers the detrimental impacts of abusive supervision on self-efficacy at work and, in turn, on creative performance. Our research not only offers implications to the literatures on abusive supervision, creative performance, mindfulness, and social cognitive theory, but also offers constructive suggestions for practice. We hope our research will inspire more endeavors to further advance our knowledge in this area in the future.

## Ethics Statement

We have obtained oral consent from all participants. The participants were informed that they were allowed to quit at any time. The study did not involve any vulnerable population and did not induce undue psychological stress.

## Author Contributions

XZ is responsible for the design of the work, revising the work critically for important intellectual content, final approval of the version to be published, and agreement to be accountable for all aspects of the work in ensuring that questions related to the accuracy or integrity of any part of the work are appropriately investigated and resolved. XL is responsible for the design of the work, analyzing the data, drafting and revising the manuscript, final approval of the version to be published, and agreement to be accountable for all aspects of the work in ensuring that questions related to the accuracy or integrity of any part of the work are appropriately investigated and resolved.

## Conflict of Interest Statement

The authors declare that the research was conducted in the absence of any commercial or financial relationships that could be construed as a potential conflict of interest.
